# Some Scientific Aspects of Insanity

**Published:** 1892-01-23

**Authors:** 


### Some Scientific Aspects of Insanity.

### IX.—ATTENTION DUE TO MANIACS.

Another form of mania, in which there is much skill and nursing power demanded on the part of the attendants is what is known as acute delirious mania. From the first there is unconsciousness, great bodily excitement and restlessness, total disregard for the calls of nature and common decency. The conversation is absolutely incoherent, the bodily temperature is usually high, rendering from 100 to 102·5, the tongue and teeth are covered with a black fur, the mouth is parched, and the eyes have a far-away, fixed appearance. As a rule, these patients have to be fed, sometimes by the attendants, sometimes forcibly by the doctor. It is extremely important accurately to observe the varying mental and bodily symptoms of this form of mania. It is necessary to note the exact quantity of food or drink that has been taken willingly or administered, to note how much time, if any, has been spent in sleep, and whether there has been even a transient return to consciousness. Above all, it is important to observe in time the first indications of failure of the bodily strength. This can often be done by the appearance of the patient indicating exhaustion, or, better still, where that is possible, by counting the number and estimating the force of the pulse beats.

The last form of mania which I have to mention is what is known as chronic mania. In medicine a disease is called chronic when it is indefinitely prolonged without any probable prospect of relief. When a patient has suffered from one or other of the different forms of mania for upwards of a year his condition is usually described as chronic mania, and at the end of that period there appears in most cases a toning down or softening of the symptoms, while the patient continues to be restless, talkative, and excited. There is less animation, less vigour, and less of freshness about his symptoms. He may continue for years in that condition of subdued excitement usually with many delusions and hallucinations. He gradually becomes less and less excited, until finally he sinks into that condition which a well-known writer on mental diseases has called the goal of all insanities, namely, secondary dementia.

The care and treatment of patients labouring under mania demands considerable attention. Mania, second only to melancholia, is the most curable of all mental diseases, and it is, therefore, demanded of us that no pains should be spared to render the chances of recovery as numerous as possible. The great aim of treatment in all cases of mania which are curable is to raise the standard of bodily health to its normal condition. It is a common mistake to suppose that a patient who is labouring under mania is stronger than a person in the full enjoyment of sound mind. The connection between mind and body is so intimate that the one cannot suffer without implicating the other, nor can the one be remedied without at the same time remedying the other. There are, therefore, three great duties which a good attendant on the insane has to perform in the treatment of mania—(1) He must exercise a healthy moral control over the patient. In order to do this the attendant himself must be healthy both in mind and body. There is no class of the insane which is so irritating, or so frequently getting both themselves and their attendants into trouble, and in order to possess a sufficient influence of a salutary nature the attendant must never lose his temper. If he does so once, the only way to regain control of the patient is by the exercise of physical force, which is brutalising and unhealthy. In order to possess a constantly equable temper the attendant must always be in good bodily health. There are no known means so powerful in the management of weak, excited, diseased minds as the influence of strong, self-possessed, firm, and sympathetic mind force. (2) It is necessary to direct into useful channels the struggling, disarranged nerve force of the maniacal patient. It is an old saying that a patient is half cured when he begins to work. The regular routine of manual labour has a most beneficial influence in restoring the disordered mental faculties. Besides work, there is amusement; there is the cheerfulness of the surroundings to be considered, the opportunity of easy access to pleasant literature, which the patient must not be allowed to destroy, and there are games, both outdoor and indoor, of every description. Now, a maniacal patient will not always and of his own accord work, or rationally amuse himself, and it is the by no means easy task of his attendant to induce him to enter upon these duties with benefit and pleasure. (3) It is necessary, above all things, that the patient should be well fed, in order to restore his bodily health to such an extent as to enable other beneficial agencies to operate upon him. An ill-fed maniacal patient will neither get well, nor will he be so quiet and free from excitement, as a well-fed patient will be. It is, therefore, necessary to observe accurately the quantity of food which he consumes, and the manner in which he eats it. It is necessary to stand over some patients while they are having food, to see that in their excitement they do not forget to eat. It is necessary to stand over others, lest in their excitement they eat their food too rapidly, or in such a manner as to endanger themselves by choking. (4) The self-respect of a patient must never, through the negligence of an attendant, be allowed to diminish. Extreme attention should be paid to those patients whose habits are faulty, both by taking precautions to prevent them from soiling their clothing, and when it is soiled, by changing it immediately, as if each occurrence were only a solitary incident in the patient's life.

---

A company is said to have been started in Washington State, U.S.A., under the title of the "Consolidated Black Cat Company," for the purpose of propagating a breed of black cats, for the sake of the fur, on the same principles which govern the breeding of cattle on Western ranches. The original stock, or the greater part of it, is to be brought from Holland, and the cats will be reared on an island, so as to prevent them having evening concerts with their brothers and sisters on the mainland. In choosing an island as the home for the cats, the promoters also doubtless had in view the desirability of protecting their undertaking from schoolboy raids and catapults. What an irresistible temptation an accessible colony of cats would be to the cruel proclivities of the ordinary schoolboy.

